# Nurses’ learning curves for ultrasound assessment of gastric content: A prospective cohort study

**DOI:** 10.1016/j.ijnsa.2026.100595

**Published:** 2026-06-07

**Authors:** Marijn C.T. Tacken, Enzio Boeijen, Reinouw Postmus-Jellema, Angela Dripal-Böinck, Alison Posthuma, Geert-Jan van Geffen, Getty Huisman-de Waal, Harm H.J. van Noort

**Affiliations:** a Department of Anesthesiology, Radboud University Medical Centre, Nijmegen, the Netherlands; b Research group Technology for Health, School of Health Studies Nijmegen, HAN University of Applied Sciences, Nijmegen, the Netherlands; c Centre for Abdominal and Pelvine Care, Radboud University Medical Centre, Nijmegen, the Netherlands; d Department of Radiology, Radboud University Medical Centre, Nijmegen, the Netherlands; e Department of IQ Health Radboud University Medical Centre, Nijmegen the Netherlands; f Department of Surgery, Radboud University Medical Centre, Geert Grooteplein Zuid 10, 6525 GA Nijmegen, Nijmegen, the Netherlands

**Keywords:** Gastric ultrasound, Nurses, Training program, Competency, Point-of-care-ultrasound (POCUS)

## Abstract

**Background:**

Gastric point-of-care ultrasonography (G-POCUS) is a novel non-invasive technique for bedside assessment of gastric content that can be performed by non-radiologic healthcare professionals, including nurses. Real-time insight into the gastric content may facilitate objective decision-making during postoperative care, such as the placement or removal of nasogastric tubes or the initiation and progression of enteral feeding. To innovate this essential aspect of nursing care, thorough implementation of G-POCUS through education is required. However, the literature remains inconclusive on the number of scans necessary for nurses to achieve competency in GPOCUS.

**Aim:**

To determine the amount of training required for nurses to achieve competence in the bedside point-of-care ultrasound for gastric content assessment.

**Methods:**

In a prospective educational cohort study**,** eight bachelor-level nurses without prior ultrasound experience enrolled in a structured training program for gastric ultrasound. They performed gastric ultrasound on healthy volunteers without abnormal upper gastrointestinal anatomy or diagnosis of diabetes. The primary outcome was to determine the number of scans a nurse requires to achieve competence in G-POCUS, defined as a 90% success rate in 10 consecutive assessments. Expert examiners served as the reference standard. Nurses were blinded to the randomized prandial status of the volunteers. Data analysis included cumulative sum scores and Bush and Mosteller’s learning model to estimate the number of examinations required to reach a 95% success rate.

**Results:**

A total of 512 assessments were conducted by eight nurses. All nurses achieved competence after a median of 23 attempts. The learning model indicated that approximately 30 scans were needed to achieve a 95% success rate.

**Conclusions:**

Structured training enables nurses to develop competency in G-POCUS, with at least 30 assessments required for skill competency when performed on healthy subjects. These findings support broader adoption of gastric ultrasound within nursing practice.

**What is already known**

• Gastric ultrasonography is a novel non-invasive technique for bedside assessment which can be performed by nurses especially because they are at the bedside 24 h a day, 7 days a week.
• The amount of training for competency in gastric ultrasound is determined for physicians, but for nurses this is unknown.

Alt-text: Unlabelled box dummy alt text

**What this paper adds**

• Structured training program in healthy volunteers for nurses for gastric ultrasound resulted in competency in all nurses
• The required number of supervised assessments is determined for a 95% success rate of nurse-performed gastric ultrasound.

Alt-text: Unlabelled box dummy alt text

## 1 Introduction

Gastric point-of-care ultrasound (G-POCUS) is a real-time bedside imaging technique performed by health care providers (Perlas et al., 2009). It enables direct visualization of gastric contents and allows estimation of gastric residual volume (GRV) by correlating it with the gastric antral cross-sectional area (Bouvet et al., 2011). Ultrasonographic measurement of gastric volume has proven to be both accurate and non-invasive, and has emerged as a promising tool across various clinical contexts (Perlas et al., 2018; Perlas et al., 2016; Van de Putte and Perlas, 2014). In perioperative care, gastric ultrasound may assist in evaluating in preoperative fasting status (van de Putte et al., 2018; Tacken et al., 2021), predicting the risk for postoperative nausea and vomiting (Wang et al., 2023), and identifying delayed bowel function (DBF) (Lamm et al., 2022). Additionally, it may support the assessment of enteral feeding tolerance in critically ill patients (Wang et al., 2022). Thus, gastric ultrasound holds potential to enhance nursing care and improve patient outcomes.

Nursing care for patients after major abdominal surgery involves the management of gastric decompression using nasogastric tubes and assessing gastric residual volume via these tubes. Patients after major abdominal surgery are at increased risk for postoperative gastro-intestinal dysfunction (Smits et al., 2022; Mazzotta et al., 2020; Sugawara et al., 2018; Zhang et al., 2024). Similarly, critically ill patients frequently experience impaired gastric and bowel motility, which can compromise enteral nutrition and necessitate regular assessment of gastric content. To support clinical decision-making regarding the need for gastric decompression, it is opportune to explore the potential contribution of G-POCUS. This may subsequently improve patient outcome and comfort. The implementation of G-POCUS in clinical practice presents an opportunity for innovation in patient care.

Introducing G-POCUS into daily practice requires a well-considered implementation strategy. Most importantly, healthcare professionals should be sufficiently skilled to obtain and interpret images. Previous studies have demonstrated learning curves for achieving competence in gastric ultrasound among anaesthesiologists and novice physicians, suggesting that a minimum of 30 assessments may be necessary (Lamm et al., 2023; Arzola et al., 2013). Some studies have described nurse training for gastric ultrasound assessments (Brotfain et al., 2022; Brunhoeber et al., 2018). These studies involved nurses performed gastric ultrasound assessments in pairs and did not specify the amount of training required to achieve independent competency. Compared to physicians, the number of scans to reach competency may plausibly differ among nurses. Hence, these professional groups differ from each other in terms of level and duration of pre-graduate education, scope of diagnostic responsibility, and prior experience in acquiring and interpreting radiologic—particularly ultrasound—images. These differences are expected to influence nurses’ learning trajectory for G-POCUS. As a result, the number of scans necessary for nurses to achieve competency in G-POCUS remains unclear.

The aim of this study was to determine the number of supervised scans a nurse requires to achieve competency in G-POCUS for the qualitative assessment of gastric content in healthy volunteers.

## 2 Methods

### 2.1 Study design

Following approval by the Medical Ethical Scientific Committee Oost-Nederland, number 2023–16,880, we conducted a prospective observational cohort study involving nurses without prior experience in ultrasonography. Under the supervision of an expert sonographer, nurses performed gastric point-of-care ultrasound (G-POCUS) on healthy volunteers. The study was conducted between March and May 2024 and adhered to the principles outlined in the Declaration of Helsinki (World Medical, 2013). It was registered at ClinicalTrials.gov (Identifier: NCT06320574) and reported in accordance with the STROBE guidelines (von Elm et al., 2014).

### 2.2 Participants

Eligible participants were bachelor-level registered nurses with a least one year of clinical experience, employed at the gastrointestinal and oncological surgery department of a university medical centre in the Netherlands. None of the participants had prior education or experience in performing ultrasonography. They may have access to view radiologic - including ultrasound – images in their role in patient care, but have no clinical responsibility in acquiring and interpreting those.

Inclusion criteria for volunteers were age 18 year or older, no history of abnormal anatomy or surgery of the upper gastrointestinal tract, and absence of the diagnosis of diabetes mellitus. Volunteers were randomised into different prandial statuses. Recruitment was conducted via social media campaigns and advertisements in supermarkets, yielding 97 respondents. Selection aimed to ensure diversity in gender, age and body mass index (BMI). All volunteers provided written informed consent and received financial compensation for their participation.

### 2.3 Teaching intervention

The training protocol was developed based on existing literature (Perlas et al., 2018; Van de Putte and Perlas, 2014; Lamm et al., 2023; Arzola et al., 2013; Brotfain et al., 2022; Yamada et al., 2023) by a multidisciplinary team compromising experts in G-POCUS and ultrasonography, a nurse-educator, and nursing researchers. Expert sonographers included an anaesthesiologist and a radiographer, each with at least 10 years’ experience in gastric or abdominal ultrasonography. General (gastric) ultrasound knowledge was tailored to nurses by incorporating basic ultrasound knowledge, including definitions, artefacts, gain, depth and penetration, and handling the ultrasound equipment.

The intervention consisted of three components.

1. Didactic module: Nurses completed an e-learning course supplemented with study materials (basics about ultrasound and review about gastric ultrasound) and a picture library (Van de Putte and Perlas, 2014; van de Putte and Bouvet; Cubillos et al., 2012).
2. Interactive session: Nurses attended a live demonstration by the expert sonographer, followed by hands-on practice performing G-POCUS on fellow trainees.
3. Supervised scanning: Nurses performed ultrasound scans on volunteers assigned to randomized prandial statuses:
  • Fasted (for at least eight hours),
  • Clear fluids (300 ml of clear lemonade, apple juice or tea with sugar),
  • Solid meal (standardized meal such as a muffin, bread, or yoghurt, accompanied by water, tea or coffee).

After each scan, nurses documented their findings and received individual feedback from the supervising examiner regarding scanning technique and image interpretation.

All scans were performed using the Mindray TE9 ultrasound system equipped with a low-frequency curved array transducer (1.0–5.0 MHz). Each examination began with the volunteer in the supine position, followed by placement into the right lateral decubitus (RLD) position. In the RLD position, fluid or semi-fluid content gravitates preferentially to the gastric antrum, enhancing antral visualization (Perlas et al., 2009). The ultrasound technique and sonographic characteristics of gastric antrum content have been described in detail elsewhere (Cubillos et al., 2012).

### 2.4 Assessment

The nurses’ competency was evaluated through direct observation and feedback from the expert sonographer following each series of ultrasound examinations. Both nurses and examiners were blinded to the prandial status of the volunteers. For examiners, prandial status could not be unblinded after the first nurse examined all patients, so examiners were only blinded during the first assessment. All nurses remained blinded for prandial status until one-to-one feedback from the examiner.

Training was conducted over five days, with both morning and afternoon sessions. In each session, three volunteers were randomly assigned to a prandial status. Volunteers underwent ultrasound in two rounds per session, with random allocation to a prandial status in each round. Short breaks every 15 min allowed volunteers to eat or drink as needed to maintain or change their prandial status.

Each volunteer was scanned twice by each participant per session. After each scan, the assigned prandial status was disclosed, and nurses received immediate one-on-one feedback from the examiner, focusing on five key areas:

1) Accurate identification of the stomach’s prandial state (fasted, clear liquid or solid food);
2) Correct acquisition of anatomical structures (antrum, liver and aorta or vena cava inferior); these anatomic landmarks must be captured in one image (Van de Putte and Perlas, 2014)
3) Technical execution of scanning procedure;
4) Accurate measurement and verification of the antral circumference in cases involving clear fluid; the circumference was estimated by drawing a line at the outside of the antral muscle in image captured between two peristaltic movements.
5) Correct estimation of gastric volume when clear fluid was present; the recommended formula for thick and clear fluids were used (Van de Putte and Perlas, 2014; Tacken et al., 2021)

Each training day concluded with a group feedback session, during which images were reviewed, and technical aspects were discussed. To ensure consistent learning progression, nurses were not allowed to practice scanning outside of scheduled sessions. However, they were encouraged to review the didactic learning material.

In the study by Arzola et al., the learning curve for anesthesiologists with prior ultrasound experience ranged between 23 and 33 scans (Arzola et al., 2013). In the present study, the number of scans per nurse was increased to 60 consecutive examinations to allow for a thorough evaluation of skill development in nurses without prior ultrasound experience. Each training included 12 scans per nurse, which were all conducted in healthy volunteers and evaluated by the expert sonographer.

### 2.5 Study endpoint

The primary study endpoint was to determine the number of scans required for nurses to achieve competency in performing gastric ultrasound. Competence was defined as achieving a success rate >90% in 10 consecutive gastric ultrasound scans. A scan was considered successful if the prandial status was correctly identified, relevant anatomical structures were accurately visualized and -if clear fluid was present- the estimated gastric volume was correct. The expert’s diagnosis served as the reference standard to the actual prandial status of the volunteers and the experts concluded whether nurses had successfully acquired and interpreted the images. Learning curves were constructed using the cumulative sum method (CUSUM) (Bould et al., 2009).

Secondary endpoints included nurses’ self-confidence in performing gastric ultrasound, assessed using the framework developed by Yamada et al. (Yamada et al., 2023), and factors that can associated with success. This framework evaluates five domains: ultrasound knowledge, anatomical knowledge, proficiency in operating ultrasound equipment, skill in acquiring gastric ultrasound images, and ability to interpret those images. Nurses rated each domain using both a 5-point Likert scale (ranging from “fully agree” to “fully disagree”), and a numeric rating scale (NRS, 1–10), with higher scores indicating greater self-confidence. The questionnaire has previously been validated by Yamada et al. (Yamada et al., 2018). Factors that may affect success included the level of nurses’ education, volunteers characteristics (including gender, age, and BMI), and gastric content based on prandial status.

### 2.6 Statistical analysis

The CUSUM graphical method was used to construct individual learning curves for each nurse to assess the primary outcome (Kestin, 1995). In CUSUM analysis, each consecutive success or failure in ultrasound assessments results in a positive or negative increment to a cumulative score. The CUSUM score increases with failure and decreases with successes, providing a visual representation of learning progression relative a predetermined standard.

Acceptable and unacceptable failure rates were defined a priori at 10%, and >30%, respectively, with α = 0.05 and β = 0.9. Competency was defined as achieving >90% correct assessments in 10 consecutive ultrasound scans. Success rate was determined as 90%, aligning with the acceptable failure rate of 10%, which was also determined in previous research on gastric ultrasound competency (Arzola et al., 2013). We determined that a failure rate greater than 30% would be unacceptable, because then a nurse would wrongly interpret images in 4 out of 10 cases (Arzola et al., 2013). Nurses were not penalized for mistakes in the beginning of the training. Subsequently, decision limits were calculated and drawn (Arzola et al., 2013; de Oliveira Filho et al., 2008). Using these limits, it would clarify whether a nurse would pass the upper line, indicating higher failure rate than accepted, or pass the under line, indicating an acceptable failure rate. A type I error threshold of 0,05 was used to determine whether nurses exceeded the statistically acceptable number of failures.

Additionally, the number of attempts required to reach competency was recorded. The expected number of scans needed to reach 90%, 95% and 99% competency levels was estimated using the Bush and Mosteller learning model (Bush and Mosteller, 1951).

Descriptive statistics were used to summarize participant characteristics and study data.

For secondary outcomes, data of factors were compared to tests differences in potential factors influencing success or failure using the independent *t*-test, Mann-Whitney test or Chi-square. A binary logistic regression analysis was conducted to determine the odds ratio’s for categorical and continuous variables. Statistical significance was set at p < .05.

All statistical analyses were performed using Microsoft Excel and using IBM SPSS Statistics (version 31) in consultation with a statistician.

## 3 Results

### 3.1 Study population

Eight nurses from the surgical nursing team participated in the study, and all completed the teaching and evaluation sessions. The median age was 27 years (range:24–48), and seven nurses were female. Five of the nurses completed a bachelor in nursing, three of the nurses completed a vocational education. The median clinical experience was 9 years (range:3–25). All nurses also volunteered to undergo G-POCUS during the initial hands-on session.

For the subsequent training days, 60 volunteers were selected from 97 respondents to the recruitment advertisements and were randomized to one of the prandial status conditions. One selected respondent did not attend the training, and this was resolved by recruiting six additional volunteers from the nursing department and the research team. In total, 74 individual healthy volunteers were included. Their mean age was 42 years (SD: 21.1; range 18–80), and the majority were female (n = 46; 62%). The mean BMI mean was 23.1kg/m^2^ (SD: 2.2).

Across all sessions, 242 prandial status conditions were evaluated in 512 individual gastric ultrasound evaluations by the nurses. During allocation 58 (24%) volunteers were assigned to the fasting regimen, 87 (36%) to the clear fluid regimen, and 97 (40%) to the solid meal condition. During evaluation, 128 (25%) were in a fasted state, 167 (33%) were filled with fluid, and 216 (42%) were in a solid food state.

### 3.2 Learning curves

A total of 512 gastric ultrasound assessments were performed by the eight nurses, with each nurse completing 64 scans (Table 1). The expert sonographer, initially blinded to the prandial status, correctly diagnosed all cases.

**Table 1:** Overall success rate and success rate per training day for the nurses (n = 8).

	Number of scans per nurse	Number of scans for all nurses (n = 8)	Successful scans of all nurses (n = 8)
Day 1	4	32	11 (34.4%)
Day 2	12	96	50 (52.1%)
Day 3	12	96	84 (87.5%)
Day 4	12	96	82 (85.4%)
Day 5	12	96	81 (84.4%)
Day 6	12	96	88 (91.7%)
Total	64	512	396 (77.3%)

**Fig. 1:**
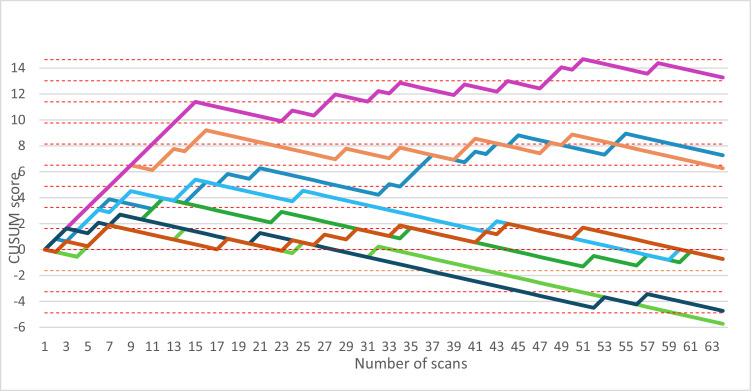
CUSUM learning curves of the nurses (n = 8) with decision limits. Red horizontal lines are decisions limits. See supplementary material for underlying data.

CUSUM analysis (Fig. 1) demonstrated that all nurses achieved competence-defined as a 90% success rate-within 64 scans. Each nurse reached the 90% successful assessments within 10 consecutive scans, thereby crossing the predefined competency threshold (Fig. 1).

The median number of scans required to reach competency was 23 with individual results of 16, 17, 17, 21, 24, 24, 25 and 29 scans.

The success rate across all assessments was 77.3% (n = 396) with individual nurses’ success rates ranging from 59% to 89%. Five of the eight nurses achieved an overall success rate exceeding 80% (81.25–89%).

**Fig. 2:**
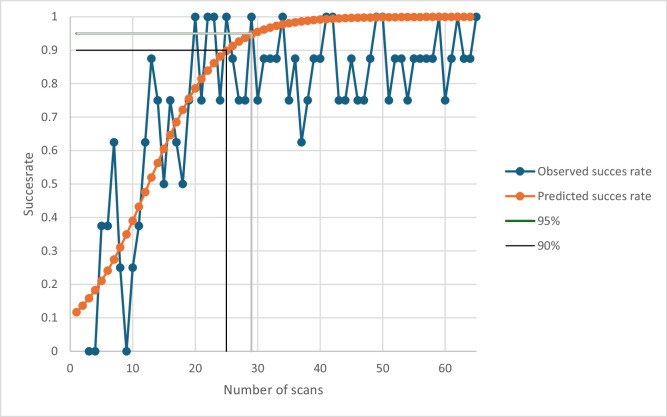
Bush and Mosteller’s predicted success rate and observed success rate. See supplementary material for underlying data.

According to the learning model by Bush and Mosteller an average of 26 scans was required to achieve a 90% success rate, 30 scans for a 95% success rate, and 39 scans for a 99% success rate (Fig. 2), see detailed predicted and observed success rates per scan in the supplementary table.

### 3.3 Nurses’ self-confidence

**Fig. 3:**
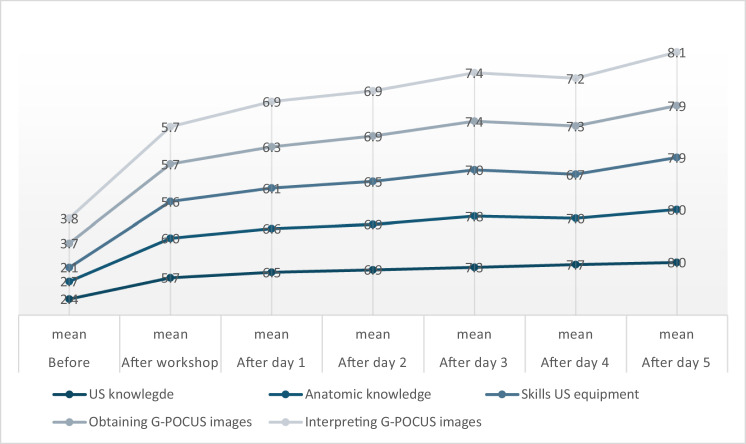
Nurses' self-confidence in performing Gastric Point-of-Care Ultrasound. G-POCUS: gastric point of care ultrasound; US= ultrasound; NRS: numeric rating scale.Numbers are mean scores (SD). NRS scores range from 1–10, 10 indicated complete self-confidence.

Self-confidence among nurses improved notably during the training period. The most substantial increase was observed in the first two days. Subsequently, nurses rated their confidence in obtaining and interpreting G-POCUS images at 7.9 (0.6) and 8.1 (0.8), respectively (Fig. 3).

### 3.4 Factors affecting success

The secondary analysis assessed whether nurses’ background or volunteers’ characteristics affected successful attempts (see Table 2). G-POCUS assessments performed by nurses with a bachelor educational level were almost three times the odds of being successful compared with those performed by nurse with vocational educational level (OR = 2.7008, 95% CI 1.77, 4.12, *p* < .001). The volunteers’ BMI was significantly higher in failure attempts. Higher BMI values were associated with lower odds of success (OR = 0.91, 95% CI 0.85, 0.98, *p* = .008). No differences were found for volunteers’ sex and age.

**Table 2:** Succes and failure rates in ultrasound assessments per educational level, volunteers’ characteristics, and gastric content.

	Succes (n = 396, 77%)	Failure (n = 116, 23%)	P-value	Odds Ratio (95% CI)
Level of nurses’ education^a^^,^*				
Vocational	127 (66)	65 (34)	<0.001#	2.70 (1.77–4.12)
Bachelor	269 (84)	51 (16)		
Volunteers				
BMI^b^	23.7 (22.0–25.3)	24.2 (22.7–27.1)	0.008$^,^&	0.91 (0.85–0.98)
Sex^a^				
Male	144 (78)	41 (22)	0.841#	(0.68–1.61)
Female	252 (77)	75 (23)		
Age (in years)	44.9 (22.2)	41.5 (20.8)	0.136@	1.01 (0.99–1.02)
Prandial status^a^			<0.001#	
Fasting	83 (21)	45 (39)		
Liquids	144 (36)	23 (20)	<0.001&	3.39 (1.92–6.00)
Solid foods	169 (43)	47 (41)	0.007&	1.95 (1.20–3.17)

⁎ = 8 nurses performed 64 assessments, 512 assessments in total;.

# =Chi-square.

$ = Mann-Whitney test;.

& = logistic regression analysis, fasting was the reference standard for prandial status;.

@ =independent student-*t*-test.

a = numbers represent number with percentages;.

b = numbers represent median with 25th and 75th quartiles. BMI: body mass index.

Gastric content based on the at random allocated prandial status showed a significant difference for success and failure attempts (*p* < .001, Chi-Square test). Compared with a fasting status, binary logistic regression analysis revealed that fluid status had more than three times the odds of success (OR = 3.39, 95% CI 1.92–6.00, *p* < .001), and solid foods status had approximately twice the odds (OR = 1.95, 95% CI 1.20–3.17, *p* = .007).

## 4 Discussion

This study aimed to determine the extent of training required for nurses to achieve sufficient competency in performing gastric point of care ultrasound (G-POCUS). All eight participating nurses reached competency after a median of 23 attempts, with an estimated 30 scans required to achieve a 95% success rate in image acquisition and interpretation. The training program was adapted from the study design of Arzola et al. (Arzola et al., 2013), who investigated training requirements for anaesthesiologists. Their findings indicated that at least 30 supervised scans were necessary to achieve competence in both qualitative and quantitative gastric ultrasound assessments.

Our program translated this approach to a nursing context, emphasizing foundational ultrasound skills, anatomical knowledge, image interpretation and structured feedback. The learning trajectory observed in our study demonstrates that nurses compared to anaesthesiologists -who reach a 95% success rate after approximately 33 scans (Arzola et al., 2013) - achieved a similar level of accuracy after 30 scans that with structured, feedback-driven training, nurses can attain a level of G-POCUS performance comparable to that of physicians in terms of qualitative and quantitative assessment.

This study contributes to the emerging knowledge on nurses performing gastric ultrasound. Previous studies have reported widely varying thresholds of supervised scans for nurses to achieve competence. Ferraboli et al., considered nurses competent after only five supervised scans (Ferraboli and Beghetto, 2022), while Gullipinar et al. used a threshold of ten scans (Güllüpinar et al., 2022). However, these thresholds were not substantiated by objective performance data or validated learning curve. Brotfain et al. described a study in which nurses performed 360 assessments in 90 ICU patients, including localisation of the gastric antrum, calculate the cross-sectional area (CSA), assess gastric residual volume, and confirm nasogastric tube positioning (Brotfain et al., 2022). Yet, the study did not clearly report whether competency was formally assessed prior to clinical deployment.

To our knowledge, our study is the first study to present a structured G-POCUS training trajectory for nurses, supported by validated learning curve methodology. These findings offer a clear benchmark for training duration and skill acquisition by nurses, with implications for nursing education and clinical implementation. Nurses and researchers must pay attention to the findings of our study when training is not performed on healthy volunteers but on clinical patients, or when selecting nurses with advanced education levels including those at intensive care units or advance practice nurses. Research must address these topics to validate the number required for competency. Moreover, it appeared that bachelor education of the nurse might affect to more success during the training. Although both vocational and bachelor nurses obtained competency, bachelor nurses may require less training compared to vocational nurses. Further research must address other confounders to optimize assessment of G-POCUS competency by nurses including work experience of nurses and inter-rater reliability of assessors.

The use of learning curves to evaluate procedural skill acquisition is increasingly prevalent in nursing research. Beyond gastric ultrasound, nurses are gaining proficiency in other ultrasound-guided procedures, such as ultrasound-guided intravenous cannulation (Loon et al., 2022). This technique improves vascular access accuracy, particularly in patients with challenging venous anatomy. Evidence suggests that structured training programs significantly improve nurses' ability to perform ultrasound-guided intravenous cannulation reducing complications and increasing first-attempt success rates (Tran et al., 2022). Such programs typically include theoretical instruction, hands-on practice, and supervised clinical application, ensuring proficiency in probe handling, vein identification, and needle guidance. The integration of ultrasound into intravenous cannulation reflects the broader use of ultrasound within nursing practice.

While our training program involved an intense schedule with healthy volunteers, future implementation of gastric ultrasound education should adopt more practical approach. Further research is needed to explore how nurses can be trained using bedside teaching, applying G-POCUS directly to patient care. Additionally, the potential role innovations in for ultrasound training might overcome the time-consuming component of training in healthy volunteers as designed in the current study. Virtual Reality (VR) is suggested to innovate ultrasound education warranting further investigation (Weiner et al., 2019; Lovquist et al., 2011; Wittek et al., 2025). VR-based training has shown promise in accelerating skill acquisition and improving image interpretation, although this may include cost and yet limited availability (Weiner et al., 2019; Lovquist et al., 2011; Wittek et al., 2025). VR and other simulation techniques enables candidates to repeat scenario’s while improving the skills, without any burden for volunteers or real patients, and provide direct feedback. Scenario’s can also represent realistic complex anatomic and pathological conditions (Wittek et al., 2025). As we found that higher BMI of the volunteers increased the odds for failure, a wide range of anatomic characterises are required.

Finally, future technologic innovation in ultrasound may further support nurses in developing competency, by facilitating the localisation of each landmark automatically when performing a gastric ultrasound.

### 4.1 Strengths and limitations

A key strength of this study is its structured and systematic approach to training. By adapting the design proposed by Arzola et al. for use among nurses, this study is the first to objectively demonstrate nurses’ ability to develop proficiency in gastric ultrasonography. Additionally, two experts participated in the evaluation process, each bringing a distinct background in ultrasonography. One with a focused procedural approach as an anaesthesiologist, and the other with a broader diagnostic perspective as a radiographer. To ensure consistency in assessment, the first training day served as a calibration session for both experts. Future research must address inter-rater reliability in supervising ultrasound assessments.

However, the study also has limitations related to its design and educational structure. Individual variation in learning progression suggests that personalized instruction may enhance training efficiency. Moreover, a temporary decline in self-confidence was observed between the 40th and 52nd assessments, potentially due to the delayed disclosure competency results until study completion. Nurses expressed a preference for receiving feedback on overall competency rather than on individual scans alone. Future training programs should therefore incorporate both per-scan feedback and cumulative performance evaluations to support learner confidence and progression.

This study also explored factors that could affect the change for success. However, the number of individual nurses are limited in this study to eight, making it hard to draw strong conclusions on whether education level affects the change for more success during the training. Especially since G-POCUS may be hard to perform in patients with higher BMI and certain prandial status. This topic, together with BMI and prandial status must be the topic in further research examining what could affect successful G-POCUS in clinical practice. In such as study, sufficient sample size on these variables must be collected to enable multivariate analysis correcting on potential confounders.

### 4.2 Implications

The study was conducted exclusively on healthy volunteers, which may not fully reflect the complexity and variability encountered in real clinical settings. Future research should investigate the validity of our findings and the feasibility of implementing a bedside training program in clinical practice, including at least 30 supervised scans on hospitalized patients. As our nurses achieved competency in healthy subjects, patients with recent surgery in the abdomen or critically ill patients may affect image acquisition and interpretation. Moreover, nurses themselves may have increased their general competency in ultrasound also affecting the number of attempts that are required. The findings of this study underscore the potential integrating gastric ultrasonography into nursing education. As point-of-care ultrasound becomes increasingly prevalent, equipping nurses with these skills may significantly enhance bedside assessment and clinical decision-making.

Further research should explore long-term skill retention, the sustainability of training outcomes, and the impact of G-POCUS on patient care in diverse clinical environments. Ultimately, this will lead to application of gastric ultrasound in routine nursing care for hospitalized patients including those after gastro-intestinal surgery

## 5 Conclusions

This study demonstrates that structured training enables nurses to achieve competency in G-POCUS, with a clearly defined number of attempts required for skill competency. By building upon the training framework established for anaesthesiologists, this research provides valuable insights into expanding gastric ultrasound education within nursing practice. Further studies should aim to refine training protocols, evaluate their clinical applicability across diverse patient populations, and explore appropriate training models for integrating G-POCUS into routine nursing care.

## Funding

The study was performed with an internal fund of the Radboud university medical centre.

## CRediT authorship contribution statement

**Marijn C.T. Tacken:** Writing – review & editing, Writing – original draft, Methodology, Formal analysis, Data curation, Conceptualization. **Enzio Boeijen:** Writing – review & editing, Supervision, Methodology, Data curation, Conceptualization. **Reinouw Postmus-Jellema:** Writing – review & editing, Project administration, Data curation, Conceptualization. **Angela Dripal-Böinck:** Writing – review & editing, Project administration, Methodology, Data curation, Conceptualization. **Alison Posthuma:** Writing – review & editing, Project administration, Data curation, Conceptualization. **Geert-Jan van Geffen:** Writing – review & editing, Supervision, Methodology, Conceptualization. **Getty Huisman-de Waal:** Writing – review & editing, Supervision, Methodology, Conceptualization. **Harm H.J. van Noort:** Writing – review & editing, Writing – original draft, Supervision, Project administration, Methodology, Funding acquisition, Formal analysis, Data curation, Conceptualization.

## Declaration of competing interest

The authors declare that they have no known competing financial interests or personal relationships that could have appeared to influence the work reported in this paper.

## Appendix Supplementary materials

Image, application 1
